# Preoperative Flexible Bronchoscopy in Patients with Persistent Ground-Glass Nodule

**DOI:** 10.1371/journal.pone.0121250

**Published:** 2015-03-24

**Authors:** Byung Woo Jhun, Sang-Won Um, Gee Young Suh, Man Pyo Chung, Hojoong Kim, O Jung Kwon, Kyung Soo Lee, Joungho Han, Jhingook Kim

**Affiliations:** 1 Division of Pulmonary and Critical Care Medicine, Department of Medicine, Samsung Medical Center, Sungkyunkwan University School of Medicine, Seoul, South Korea; 2 Department of Radiology and Center for Imaging Science, Samsung Medical Center, Sungkyunkwan University School of Medicine, Seoul, South Korea; 3 Department of Pathology, Samsung Medical Center, Sungkyunkwan University School of Medicine, Seoul, South Korea; 4 Department of Thoracic and Cardiovascular Surgery, Samsung Medical Center, Sungkyunkwan University School of Medicine, Seoul, South Korea; Fundación Jimenez Diaz, SPAIN

## Abstract

There are no accurate data on the diagnostic value of preoperative flexible bronchoscopy (FB) for persistent ground-glass nodule (GGN) of the lung. We evaluated the value of preoperative FB in patients with suspected GGN-type lung cancer. We retrospectively searched a database for subjects who had ‘ground-glass opacity’, ‘non-solid nodule’, ‘part-solid nodule’, or ‘sub-solid nodule’ on chest computed tomography reports between February 2004 and March 2012. Patients who had infiltrative ground-glass opacity lesions, mediastinal lymphadenopathy, or pleural effusion, focal ground-glass opacity lesions >3 cm, and were lost to follow-up were excluded. We assessed the diagnostic value of preoperative FB in patients with persistent GGNs who underwent surgical resection. In total, 296 GGNs were evaluated by FB in 264 patients with persistent GGNs who underwent preoperative FB and surgical resection. The median size of the GGNs was 18 mm; 135 (46%) were pure GGN and 161 (54%) were part-solid GGN. No visible tumor or unsuspected endobronchial metastasis was identified by preoperative FB. Only 3 (1%, 3/208) GGNs were identified preoperatively as malignant by bronchial washing cytology; all were part-solid GGNs. No other etiology was identified by FB. Of the GGNs, 271 (91%) were subsequently confirmed as malignant and 25 (9%) were confirmed as benign at surgical resection. Consequently, the overall diagnostic sensitivity and negative predictive value of preoperative FB on a per-nodule basis was 1% (3/271) and 8% (25/293), respectively. The preoperative FB did not change the surgical strategy. Preoperative FB did not add much to the evaluation of persistent GGNs of the lung. Routine preoperative FB may have limited value in surgical candidates with small persistent pure GGNs.

## Introduction

Pulmonary nodules are a common, worrying clinical problem because they often indicate early-stage lung cancer [1, 2]. As a low-dose chest computed tomography (CT) screening trial showed a 20% mortality reduction, the management of pulmonary nodules has become an important issue for clinicians with the increased use of chest CT [3]. Recent guidelines for the management of pulmonary nodules have recommended strategies that include observation with serial radiographs, bronchoscopic or transthoracic needle biopsies, and surgical resection [4]. However, choosing the appropriate strategy is difficult because several factors can influence the diagnostic values of the strategies [5].

Persistent ground-glass nodules (GGNs) can indicate focal fibrosis, premalignant lesions, or subtypes of adenocarcinoma [6–8], and an early confirmatory diagnosis without surgery is difficult [9]. Follow-up for more than 2 years is needed for GGN-type lung adenocarcinoma due to the long doubling time [10, 11]. A bronchoscopic biopsy is usually impossible for GGN-type lung adenocarcinoma because endobronchial metastasis is uncommon and most lesions arise in peripheral areas [12–14]. Non-diagnostic transthoracic needle biopsy results cannot rule out the possibility of a malignancy [4]. Therefore, surgical resection with diagnostic and curative intent is usually performed for persistent GGNs that suggest malignancy.

Before the surgical resection of non-small cell lung cancer (NSCLC), flexible bronchoscopy (FB) is performed to identify the underlying etiology, unsuspected endobronchial involvement, and anatomical variation. However, the value of FB in the routine preoperative work-up of pulmonary nodules is still controversial. The American College of Chest Physicians guidelines do not recommend routine preoperative FB for indeterminate small pulmonary nodules [4, 15, 16], however, recent studies [17, 18] have demonstrated the usefulness of preoperative FB, including the ability of FB to identify unsuspected endobronchial involvement and change the planned surgical approach.

These contradictory reports can be explained in part by the fact that several factors influence the diagnostic yield of FB, including the size and location of the nodules and the prevalence of malignancy [4, 9, 13, 15]. However, few reports on the diagnostic value of preoperative FB for pulmonary nodules focus on the radiological characteristics of the nodules [19, 20]. Therefore, this study retrospectively evaluated the value of preoperative FB in patients with suspected GGN-type lung cancer who underwent planned surgical resection with diagnostic and curative therapeutic intent.

## Materials and Methods

### Study subjects and data collection

We searched a database for subjects treated at Samsung Medical Center, a 1,961-bed referral hospital in Seoul, South Korea, between February 2004 and March 2012 who had ‘ground-glass opacity’, ‘non-solid nodule’, ‘part-solid nodule’, or ‘sub-solid nodule’ in their chest CT report and reviewed their medical records.

Data on patient characteristics, chest CT and positron emission tomography (PET)/CT findings of GGNs, preoperative FB findings, results of preoperative diagnostic evaluations (bronchial washing cytology, transbronchial lung biopsy, and transthoracic needle biopsy), histopathology, and patient outcomes were collected. Follow-up data were last obtained on December 31, 2012.

This study was approved by the Institutional Review Board of Samsung Medical Center (IRB No. 2012–04–059). The requirement for informed consent from the individual patients was waived given the retrospective nature of the study.

### Radiological evaluation of GGNs

A GGN was defined as a rounded area of homogeneous or heterogeneous increased attenuation on CT with a lower density than surrounding soft-tissue structures, such as vessels [21]. The GGNs were subclassified as either pure or part-solid GGN [7, 21, 22]. Both mediastinal (width, 400 Hounsfield units [HU]; level, 20 HU) and lung (width, 1,500 HU; level, –700 HU) window CT images of the GGNs were viewed and the definitions of pure and part-solid GGNs were based on the tumor shadow disappearance rate (TDR): solid (TDR = 1), part-solid (0 < TDR < 1), and pure (TDR = 0) GGN [23]. Nodules were considered peripherally distributed if they were located in the lateral two-thirds of the lung parenchyma on transverse CT. The number, size (diameter), characteristics (pure or part-solid), margin (smooth or spiculated/lobulated), distribution (central or peripheral), location, and other abnormal findings of the GGNs were evaluated.

### Preoperative FB and diagnostic evaluation

A preoperative FB (EVIS BF 1T240 and 1T260; Olympus; Tokyo, Japan) evaluation of the bronchial tree was performed by experienced pulmonologists. All procedures were performed under conscious sedation with midazolam. Local anesthesia was achieved by nebulization with 4% lidocaine. When no endobronchial lesion was visible through the bronchoscope, bronchial washing was performed in the corresponding segmental bronchus. The bronchial washing was performed using 10–20 mL of saline; when the volume of fluid recovered was insufficient, an additional 10-mL saline was injected. The washing aspirates were sent for cytological examination and staining or microbiological culture for bacteria and fungi. A transbronchial lung biopsy or bronchoscopic mucosal biopsy was performed when there was a suspicious lesion, and a transthoracic needle biopsy was performed at the physician’s discretion.

All samples were categorized based on the cyto-histopathological reports. The presence of frank malignant cells or rare cells suspicious for malignancy was considered positive. The absence of tumor cells and dysplastic bronchial epithelium and the presence of normal bronchial epithelial cells was considered negative. Surgically confirmed adenocarcinoma was classified according to the new lung adenocarcinoma classification of the European Respiratory Society / International Association for the Study of Lung Cancer / American Thoracic Society [24]. All specimens were evaluated by an experienced lung pathologist (JH). The gold standard for the final diagnosis of the FB was based on the pathology of the surgical specimen.

### Statistical analysis

All data are presented as medians (interquartile range [IQR]) for continuous variables and numbers (percentage) for categorical variables. The diagnostic sensitivity, specificity, positive predictive value (PPV), and negative predictive value (NPV) of preoperative FB were calculated on a per-nodule basis. Data were compared using the Mann–Whitney *U*-test for continuous variables and the chi-square or Fisher’s exact test for categorical variables. All statistical analyses were performed using PASW 18.0 (SPSS, Chicago, IL) and a two sided *p* < 0.05 was considered to indicate statistical significance.

## Results

### Characteristics of the study patients

**Fig 1:**
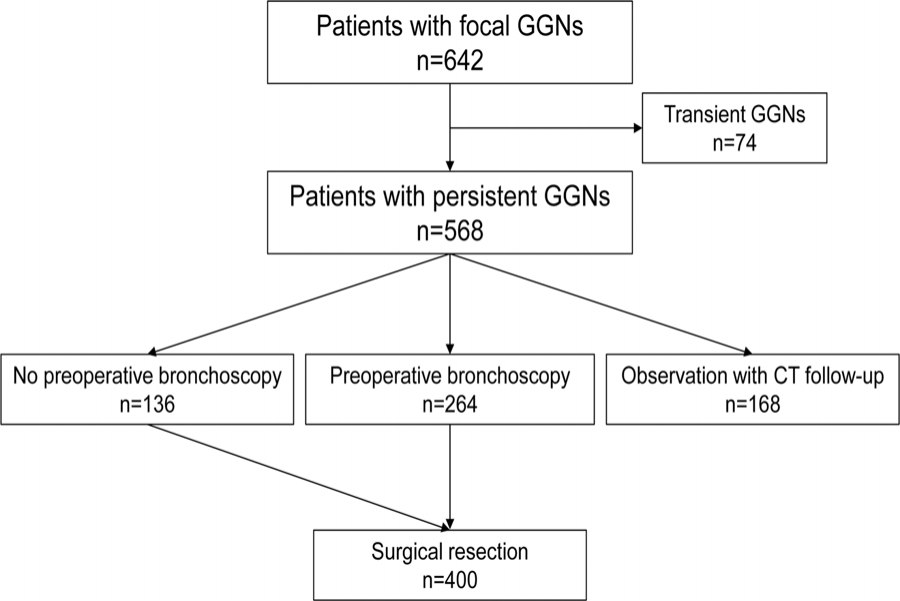
Diagram of the management for patients with a GGN on chest computed tomography.

The database search for subjects who had ‘ground-glass opacity’, ‘non-solid nodule’, ‘part-solid nodule’, or ‘sub-solid nodule’ on chest CT identified 984 patients. From these, we excluded patients with infiltrative ground-glass opacity lesions, mediastinal lymphadenopathy, or pleural effusion (n = 251), focal ground-glass opacity lesions > 3 cm (n = 18), and those lost to follow-up (n = 73). Consequently, 642 patients with focal GGNs were identified. Of these 642, patients with transient GGNs (n = 74), patients who did not undergo preoperative FB (n = 136), and patients on observation with CT follow-up (n = 168) were excluded. Ultimately, 264 patients with persistent GGNs who underwent preoperative FB and surgical resection were included in the study (Fig. 1).

The clinical characteristics of the 264 study patients are summarized in Table 1. The median age was 59 years (IQR 53–64 years), 124 (47%) were male, and 177 (67%) had never smoked. Nineteen (7%) patients had previous extrathoracic malignancies, with thyroid cancer being the most common (n = 8, 3%). Most (n = 231, 81%) patients had a single GGN, and 51 (19%) patients had multiple GGNs.

**Table 1:** Characteristics of the study patients.

Characteristics	
Patients	264 (100)
Age (years)	59 (53–64)
Sex (male / female)	124 (47) / 140 (53)
Smoking	
Never smoker	177 (67)
Ex-smoker	53 (20)
Current smoker	34 (13)
Underlying disease	
Previous extrathoracic malignancy	19 (7)
Thyroid cancer	8 (3)
Colorectal cancer	5 (2)
Breast cancer	3 (1)
Stomach cancer	3 (1)
Previous tuberculosis	5 (2)
Interstitial lung disease	2 (1)
Number of GGNs per patients	
1	213 (81)
2	41 (15)
≥ 3	10 (4)
Preoperative diagnostic evaluation	
Bronchoscopic inspection	264 (100)
Bronchial washing cytology	194 (74)
Bronchoscopic mucosal biopsy	4 (2)
Transbronchial lung biopsy	2 (1)
Transthoracic needle biopsy	58 (22)
Histopathological diagnosis	
Non-small cell lung cancer	249 (94)
T1aN0M0	147 (55)
T1aN1M0	2 (1)
T1bN0M0	85 (32)
T1bN1M0	3 (1)
T2aN0M0	11 (4)
T2aN1M0	1 (1)
Benign lung disease	15 (6)
Treatment	
Surgical resection alone	254 (96)
Surgical resection with adjuvant chemotherapy	10 (4)
Clinical follow-up period, months	40 (23–57)

Data are shown as medians (interquartile range) or no. (%).

GGN, ground-glass nodule; NSCLC, non-small cell lung cancer.

Bronchial washing cytology (n = 194, 74%), a bronchoscopic mucosal biopsy (n = 4, 2%), or a transbronchial lung biopsy (n = 2, 1%) was performed during preoperative FB. A transthoracic needle biopsy was performed preoperatively in 58 (22%) patients. Most (n = 249, 94%) patients had malignant nodules, with NSCLC T1aN0M0 being the most common (n = 147, 55%), and 15 (6%) patients had benign nodules. The median duration of clinical follow-up was 40 months (IQR 23–57 months), and 3 (1%) patients died during follow-up period due to pneumonia (n = 1), recurrent lung cancer (n = 1), and an unknown cause (n = 1).

### Characteristics of GGNs

In total, 296 GGNs were evaluated by FB in 264 patients during the study period. The characteristics of the GGNs are summarized in Table 2. The median size was 18 mm (IQR 2–22 mm), 135 (46%) were pure GGNs, and 161 (54%) were part-solid GGNs. Approximately half of all nodules (n = 142, 48%) had smooth margins and 89% (n = 262) were distributed peripherally. An air bronchogram was observed in 29 (10%) patients and bubble lucency was observed within the GGN in 19 (7%) patients. PET/CT was available for 278 nodules; only 21% (59/278) had high uptake (SUV_max_ ≥ 2.5).

**Table 2:** Characteristics of GGNs included in the analysis.

Characteristics	
Number of GGNs	296 (100)
Chest CT findings	
Size (mm)	18 (12–22)
Characteristics	
Pure GGN	135 (46)
Part-solid GGN	161 (54)
Margin of nodules	
Smooth	142 (48)
Spiculated or lobulated	154 (52)
Distribution of nodules	
Central	34 (11)
Peripheral	262 (89)
Location	
Right upper lobe	108 (37)
Right middle lobe	12 (4)
Right lower lobe	65 (22)
Left upper lobe	75 (25)
Left lower lobe	36 (12)
Air bronchogram	29 (10)
Bubble lucency	19 (7)
Histopathological diagnosis	
Malignancy	271 (91)
Invasive adenocarcinoma	172 (58)
Minimally invasive adenocarcinoma	29 (10)
Adenocarcinoma *in situ*	70 (23)
Benign	25 (9)
Chronic inflammation	14 (5)
Granulomatous inflammation	8 (3)
Atypical adenomatous hyperplasia	3 (1)
GGNs with PET/CT up take (SUV_max_≥2.5)	59/278 (21)

Data are shown as medians (interquartile range) or no. (%).

GGN, ground-glass nodule; PET, positron emission tomography; CT, computed tomography; SUV_max_, maximum standardized uptake.

Of the GGNs, 271 (91%) were confirmed as malignant with the surgical specimen, and these included invasive adenocarcinoma (n = 172, 58%), minimally invasive adenocarcinoma (n = 29, 10%), and adenocarcinoma *in situ* (n = 70, 23%). The remaining 25 (9%) GGNs were confirmed as benign, and included chronic inflammation (n = 14, 5%), granulomatous inflammation (n = 8, 3%), and atypical adenomatous hyperplasia (n = 3, 1%). The median duration from hospital presentation to surgical resection was 5 months (IQR 4–7 months).

### Results of diagnostic evaluations

The results of the diagnostic evaluations are summarized according to the characteristics of the GGNs in Table 3. Of the GGNs, 135 (46%) pure GGNs and 161 (54%) part-solid GGNs were evaluated preoperatively by FB. The median size of the part-solid GGNs was significantly larger than the pure GGNs (*p* < 0.001). Of the 135 pure GGNs, 89% (n = 120) were malignant, adenocarcinoma *in situ* being the most common (n = 57, 42%), and of the 161 part-solid GGNs, 94% (n = 151) were malignant, invasive adenocarcinoma being the most common (n = 129, 80%).

**Table 3:** Results of diagnostic evaluation according to characteristics of GGNs.

Characteristics	Pure GGN	Part-solid GGN	*p*-value
Number of GGNs	135	161	
Size (mm)	13 (10–18)	20 (16–25)	<0.001
Bronchoscopic inspection			0.420
Anthracofibrosis	4/135 (3)	9/161 (5)	
Narrowing or stricture	2/135 (1)	2/161 (1)	
Mucosal irregularity	0/135 (0)	3/161 (2)	
Nodular lesion	2/135 (1)	1/161 (1)	
No endobronchial lesion	127/135 (95)	146/161 (91)	
Bronchial washing cytology			0.269
Positive for malignancy	0/86 (0)	3/122 (2)	
Bronchoscopic mucosal biopsy			NA
Positive for malignancy	0/2 (0)	0/3 (0)	
Transbronchial lung biopsy			NA
Positive for malignancy	0 (0)	0/3 (0)	
Transthoracic needle biopsy			0.698
Positive for malignancy	6/8 (75)	32/52 (62)	
Histopathological diagnosis			<0.001
Malignancy	120/135 (89)	151/161 (94)	
Invasive adenocarcinoma	43/135 (32)	129/161 (80)	
Minimally invasive adenocarcinoma	20/135 (15)	9/161 (6)	
Adenocarcinoma *in situ*	57/135 (42)	13/161 (8)	
Benign	15/135 (11)	10/161 (6)	
Chronic inflammation	10/135 (7)	4/161 (2)	
Granulomatous inflammation	3/135 (2)	5/161 (3)	
Atypical adenomatous hyperplasia	2/135 (2)	1/161 (1)	

Data are presented as the medians (interquartile range) or no. (%).

GGN, ground-glass nodule; NA, not applicable.

Overall, no visible tumor or unsuspected endobronchial metastasis was identified by preoperative FB, and only a few benign lesions were observed (13 anthracofibrosis, four narrowings or strictures, three mucosal irregularities, and three nodular lesions). Bronchial washing cytology was performed in 208 of the 296 GGNs: 122 part-solid GGN and 86 pure GGN. Only 3 (1%, 3/208) GGNs were identified as malignant preoperatively, all of which were part-solid GGN; no other accompanying etiology was identified by FB. The results of bronchoscopic mucosal biopsies performed on two nodular lesions and three mucosal irregular lesions were all negative. Transbronchial lung biopsies were available only for three part-solid GGNs and all were negative. Consequently, the overall diagnostic sensitivity, specificity, PPV, and NPV of preoperative FB for GGN identification on a per-nodule basis were 1% (3/271) 100% (25/25), 100% (3/3), and 8% (25/293), respectively. Routine preoperative FB did not change the planned surgical strategy in any patient.

Table 4 presents the characteristics of the patients who were diagnosed with malignancy by FB preoperatively. All three patients had part-solid GGNs larger than 20 mm with speculated or lobulated margins, and an air bronchogram (n = 2) or bubble lucency (n = 1) was observed. However, there were no unsuspected endobronchial lesions on FB.

**Table 4:** Characteristics of patients preoperatively diagnosed with malignancy by preoperative bronchoscopy.

Characteristics	CASE 1	CASE 2	CASE 3	
Age (year)	59	63	76	
Sex	Female	Female	Female	
Smoking	Never	Never	Never	
Number of GGNs per patients	1	1	2	
Chest CT findings				
Size (mm)	28	28	25	19
Characteristics	Part-solid	Part-solid	Part-solid	Pure
Margin	Spiculated	Lobulated	Spiculated	Smooth
Distribution	Peripheral	Peripheral	Peripheral	Peripheral
Location	Right lower lobe Superior segment	Right upper lobe Posterior segment	Right upper lobe Posterior segment	Right upper lobe Apical segment
Air bronchogram	No	Yes	Yes	No
Bubble lucency	Yes	No	No	No
PET/CT up take (SUV_max_≥2.5)	Yes	Yes	Yes	No
Bronchoscopic inspection	No lesion	No lesion	Anthracofibrosis	
Bronchial washing cytology	Positive	Positive	Positive	Negative
Histopathological diagnosis	Invasive adenocarcinoma	Invasive adenocarcinoma	Invasive adenocarcinoma	Minimally invasive adenocarcinoma
Surgical stage	T1bN0M0	T1bN0M0	T1bN0M0	T1aN0M0

Data are presented as the medians (interquartile range) or no. (%).

GGN, ground-glass nodule; CT, computed tomography; PET, positron emission tomography; SUV_max_, maximum standardized uptake; NA, not applicable.

## Discussion

In this study, no visible tumor or unsuspected endobronchial metastasis was identified by preoperative FB in patients with persistent GGNs. Only three part-solid GGNs were identified preoperatively as malignant by bronchial washing cytology, despite the high incidence of malignancy (271/296, 92%); no other accompanying etiology was identified. Consequently, the overall diagnostic sensitivity and NPV of preoperative FB for GGN identification on a per-nodule basis were only 1% (3/271) and 8% (25/293), respectively, and preoperative FB did not change the planned surgical strategy in any patient. Moreover, given that there was no unsuspected endobronchial metastasis and no need to change the surgical approach in 136 patients with persistent GGNs who underwent surgical resection without preoperative FB, in whom 123 (90%) had NSCLC IA, 2 (2%) had NSCLC IB, and 11 (8%) had benign lung diseases, our data suggest that routine preoperative FB may have limited value in surgical candidates with small persistent pure GGNs.

These results are supported by previous reports that persistent GGNs occasionally indicate benign lung diseases [8] and GGN-type lung cancer shows minimal invasive growth with relatively long doubling times [10] and a better prognosis than the solid type [10, 11, 14, 25–27]. For example, in a study that evaluated 61 patients with primary lung cancer who underwent 3-year mass CT screening, Hasegawa *et al*.[10] reported that atypical adenomatous hyperplasia and GGN-type adenocarcinomas had greater volume-doubling times than squamous cell carcinomas. Asamura *et al*.[27] compared the characteristics of 28 GGN and 20 solid-type lung cancer cases and found no nodal metastasis in the GGN-type lung cancers in contrast to the solid-type cancers, and the 5-year disease-free survival rate was 100% for GGN-type lung cancer.

In this context, our data imply that the diagnostic value of FB for persistent lung nodules is dependent on the characteristics of the nodule, not merely its size or location [4, 9, 13, 15], and diagnostic strategies might be adjusted according to the presence of a ground-glass opacity. Other studies of the diagnostic yield of FB for lung cancer have reported that the sensitivity of bronchoalveolar lavage or washing cytology was 29–78% for central lung lesions and 12–65% for peripheral lung lesions [28], which is considerably higher than in our work; however, those studies did not mention the characteristics of the lesions. This might explain the contradictory results regarding the diagnostic value of FB across studies and suggest that discrimination between GGNs and solid-type nodules is important, since this distinction could eventually affect diagnostic strategies. To date, however, only limited data exist on the value of FB for persistent GGNs compared to solid nodules. Further studies focusing on the characteristics of the nodules are needed.

In this study, we also evaluated difference in the diagnostic value of FB between pure and part-solid GGNs (Table 3) and found that all three GGNs identified preoperatively as malignant by FB were part-solid GGNs, suggesting a relatively lower diagnostic value of FB for pure GGNs compared to part-solid GGNs. However, there was no significant difference in the results of the diagnostic evaluation, except for nodule size, and these results might have resulted from the fact that the overall diagnostic sensitivity of FB for GGNs was very low in our study and only a small proportion of patients underwent aggressive bronchoscopic procedures, such as a transbronchial lung biopsy or mucosal biopsy.

This study had several limitations. First, because the study patients were selected retrospectively based only on chest CT reports, the data should be interpreted conservatively. Second, not all GGNs were subject to bronchoscopic washing cytology, and only a small proportion of patients underwent aggressive bronchoscopic procedures without using guidance, such as CT or fluoroscopy [29]. Therefore, it is possible that the diagnostic value of FB was underestimated. For more accurate analysis, further prospective studies of the value of preoperative FB using various diagnostic techniques in suspected persistent GGNs may be needed.

## Conclusions

Preoperative FB did not add much to the evaluation of persistent GGNs of the lung. Routine preoperative FB may have limited value in surgical candidates with small persistent pure GGNs.
